# Use of a novel antigen expressing system to study the *Salmonella enterica* serovar Typhi protein recognition by T cells

**DOI:** 10.1371/journal.pntd.0005912

**Published:** 2017-09-05

**Authors:** Rosângela Salerno-Gonçalves, Hervé Tettelin, David Lou, Stephanie Steiner, Tasmia Rezwanul, Qin Guo, William D. Picking, Vishvanath Nene, Marcelo B. Sztein

**Affiliations:** 1 Center for Vaccine Development (CVD), Department of Pediatrics, University of Maryland School of Medicine, Baltimore, MD, United States of America; 2 Department of Microbiology and Immunology and Institute for Genome Sciences (IGS), University of Maryland School of Medicine, Baltimore, MD, United States of America; 3 Department of Pharmaceutical Chemistry, University of Kansas, Lawrence, Kansas, United States of America; 4 International Livestock Research Institute (ILRI), Nairobi, Kenya; Institut Pasteur, FRANCE

## Abstract

*Salmonella enterica* serovar Typhi (*S*. Typhi), the causative agent of the typhoid fever, is a pathogen of great public health importance. Typhoid vaccines have the potential to be cost-effective measures towards combating this disease, yet the antigens triggering host protective immune responses are largely unknown. Given the key role of cellular-mediated immunity in *S*. Typhi protection, it is crucial to identify *S*. Typhi proteins involved in T-cell responses. Here, cells from individuals immunized with Ty21a typhoid vaccine were collected before and after immunization and used as effectors. We also used an innovative antigen expressing system based on the infection of B-cells with recombinant *Escherichia coli* (*E*. *coli*) expressing one of four *S*. Typhi gene products (i.e., SifA, OmpC, FliC, GroEL) as targets. Using flow cytometry, we found that the pattern of response to specific *S*. Typhi proteins was variable. Some individuals responded to all four proteins while others responded to only one or two proteins. We next evaluated whether T-cells responding to recombinant *E*. *coli* also possess the ability to respond to purified proteins. We observed that CD4^+^ cell responses, but not CD8^+^ cell responses, to recombinant *E*. *coli* were significantly associated with the responses to purified proteins. Thus, our results demonstrate the feasibility of using an *E*. *coli* expressing system to uncover the antigen specificity of T-cells and highlight its applicability to vaccine studies. These results also emphasize the importance of selecting the stimuli appropriately when evaluating CD4^+^ and CD8^+^ cell responses.

## Author summary

*Salmonella enterica* serovar Typhi (*S*. Typhi) is the causative agent of the life-threatening typhoid fever that affects 11.9–20.6 million individuals annually in low-income and middle-income countries. The T-cells, CD4^+^ and CD8^+^ T cells, play a significant role in protection against *S*. Typhi infection. Yet, the antigens triggering host protective immune responses recognized by these cells are largely unknown. To address this shortcoming, in this study we used an *E*. *coli* expression system methodology for identifying immunogenic proteins of *S*. Typhi. We found that although the pattern of response to individual *S*. Typhi proteins was variable among the typhoid vaccinees, the *E*. *coli* expressing system uncovered the antigen specificity of T-cells, and highlight its applicability to vaccine studies.

## Introduction

Typhoid fever is caused by *Salmonella enterica* serovar Typhi (*S*. Typhi), a human-restricted pathogen that enters the host through the gut-associated lymphoid tissue. Recent calculations of the typhoid burden estimated that 11.9–20.6 million new cases of typhoid fever occur annually in low-income and middle-income countries with about 129,000–223,000 mortality [1–4]. Based on data provided by the World Health Organization, 90 percent of these typhoid deaths occur in Asia, and most victims are children under five years of age [5]. Furthermore, antimicrobial treatment of enteric fever and asymptomatic carriers has become increasingly complicated due to the emergence of multidrug-resistant strains of *S*. Typhi [6–8]. Thus, there has been an increased emphasis on control measures, such as vaccination to fight *S*. Typhi infection [9, 10]. It has also become evident that a better understanding of the host immune responses against *S*. Typhi will be required to achieve this task. Currently, two typhoid vaccines are licensed in the USA for use in humans, the purified Vi (“virulence”) parenteral polysaccharide vaccine and the oral live attenuated *S*. Typhi strain Ty21a vaccine. Although these vaccines are moderately protective and able to induce herd immunity [11, 12], they also have some significant shortcomings. Since Vi is a T-cell independent antigen, Vi vaccine does not confer “memory,” and there are no robust data to suggest that the efficacy of Vi persists beyond three years [11, 13, 14]. The Ty21a vaccine, which does not elicit anti-Vi antibodies, requires the administration of three to four doses spaced at 48-hour intervals [12, 13, 15]. Moreover, recently, Vi-protein-conjugate vaccines that consist of the *S*. Typhi Vi polysaccharide covalently bound to a carrier protein have been developed [5, 16–19]. However, issues have been raised about selective pressure for the development and spread of *S*. Typhi Vi antigen-negative strains due to the generalized use of Vi and Vi-protein-conjugate vaccines [20, 21]. As a result, novel approaches to typhoid vaccination are critically needed [22].

It is now widely accepted that cellular-mediated immunity (CMI) plays a significant role in protection against *S*. Typhi infection [8]. These host responses rely mainly on two types of T-cells, CD4^+^ and CD8^+^ T cells [23–26]. The presence of both CD4^+^ helper T-cells and classical class Ia and non-classical HLA-E-restricted *S*. Typhi-specific CD8^+^ T cells have been observed in individuals who recover from typhoid fever [25] or immunized with Ty21a and other attenuated leading typhoid vaccine candidates, including CVD 906, CVD 908, CVD 908-*htrA* and CVD 909 [26–33]. Moreover, our group recently provided the first evidence that *S*. Typhi-specific CD8^+^ responses correlate with clinical outcome in humans challenged with wild-type *S*. Typhi [34]. However, the antigen specificity of these T cells remains largely unknown. Moreover, most of the *S*. Typhi proteins described as being involved in human protection have been derived from studies using mouse models of *Salmonella* infection [35, 36]. One of the reasons for this is the inherent problems of working with humans as experimental models.

Here, we used an innovative antigen expressing system, originally developed by the Higgins laboratory [37, 38] and based on the infection of B-cells with recombinant *E*. *coli* to evaluate T cell responses to four *S*. Typhi proteins: SifA, FliC, GroEL, and OmpC (**Table 1**). These proteins are known to confer survival properties to *Salmonella* and therefore might be evaluated as vaccine antigens [27, 39–44]. Briefly, in this system, EBV-transformed lymphoblastoid B-cell lines (B-LCL) were used as antigen-presenting cells (APCs). These B-LCL were infected with *E*. *coli* expressing both *S*. Typhi proteins and cytoplasmic listeriolysin O (Hly). Hly is a pore-forming hemolysin from *Listeria monocytogenes*, which allows leakage of *E*. *coli* antigen from the phagolysosomal compartment into the APC cytosol, there gaining access to the MHC class I antigen processing and presentation pathway [37, 38]. This system also allows the identification of *S*. Typhi-specific CD4^+^ T cell as the expression on *E*. *coli* also results in antigen presentation in the context of MHC class II molecules [45]. Additionally, this approach has the advantage of assessing T-cell responses to full-length proteins before initiating more expensive and time-consuming procedures, such as synthesizing overlapping peptides [46]. Due to HLA diversity in humans, host responses to subunit vaccines have a greater chance to be successful if they encompass specific protein antigens rather than specific epitopes within those proteins [45, 46].

**Table 1:** *S*. Typhi proteins evaluated in this manuscript.

Protein Name	Gene Name	Function	MW (kd)
Putative virulence determinant	SifA	Required for endosomal tubulation and formation of Salmonella-induced filaments (Sifs)	38.6
Flagellin	FliC	Bacterial-type flagellum-dependent cell motility	53.2
Chaperonin	GroEL	Promotes protein refolding	57.2
Outer membrane protein C	OmpC	Forms pores that allow passive diffusion of small molecules across the outer membrane	41.2

By using this innovative antigen expression system, we found that the pattern of response to individual *S*. Typhi proteins was variable. Some individuals responded to all four proteins while others responded to only one or two proteins. When comparing T cells responses to B-LCL exposed to recombinant *E*. *coli* to those to purified proteins from the same genes, we observed that the CD4^+^ cell responses, but not CD8^+^ cell responses, to recombinant *E*. *coli* were significantly associated with the responses to purified proteins. Thus, our results demonstrate the feasibility of using an *E*. *coli* expressing system to uncover the antigen specificity of T-cells, and highlight its applicability to vaccine studies. These results also emphasize the importance of selecting the stimuli appropriately when designing experiments aimed at evaluating CD4^+^ and CD8^+^ cell responses.

## Results

### Expression of recombinant proteins

**Fig 1:**
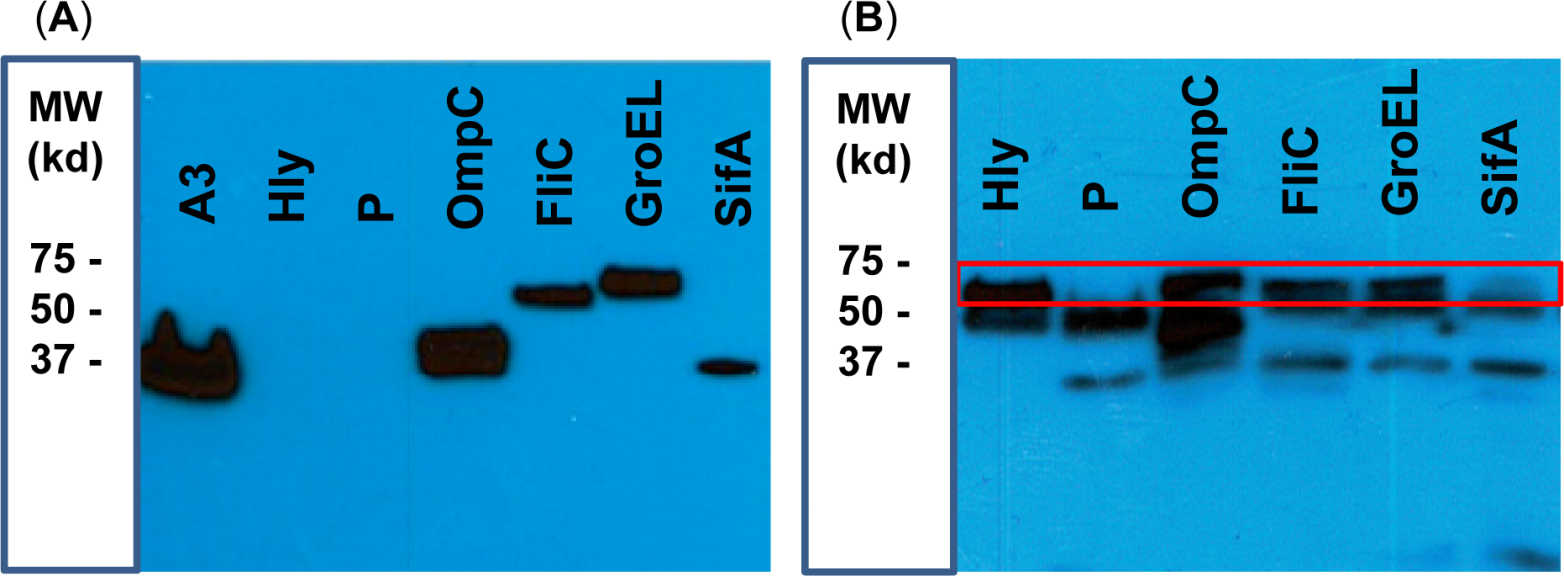
Expression of *S*. Typhi proteins and lysteriolysin on recombinant *E*. *coli*. (**A**) Anti-HisTag antibody revealing positive control protein Annexin3 (A3) as well as lysteriolysin (Hly) and *S*. Typhi gene encoded proteins (OmpC, FliC, GroEL and SifA). A negative control with pmark (P, stop codons) clones was used instead of the protein. (**B**) Anti-lyteriolysin antibody revealing Hly gene expression (red box) in all lanes except in the negative control. Expression of *S*. Typhi proteins and lysteriolysin on recombinant *E*. *coli* were detected by Western blot.

To show the feasibility of our *E*. *coli* expressing system, we evaluate four *S*. Typhi proteins (i.e., SifA, FliC, GroEL, and OmpC) (**Table 1**) known to confer survival properties to *Salmonella* then potentially promising as vaccine antigens [27, 39–44]. As shown in **Fig 1**, proper *E*.*coli* protein expression for all four proteins, SifA, OmpC, FliC, and GroEL, as well as the Hly was detected by Western blot.

**Fig 2:**
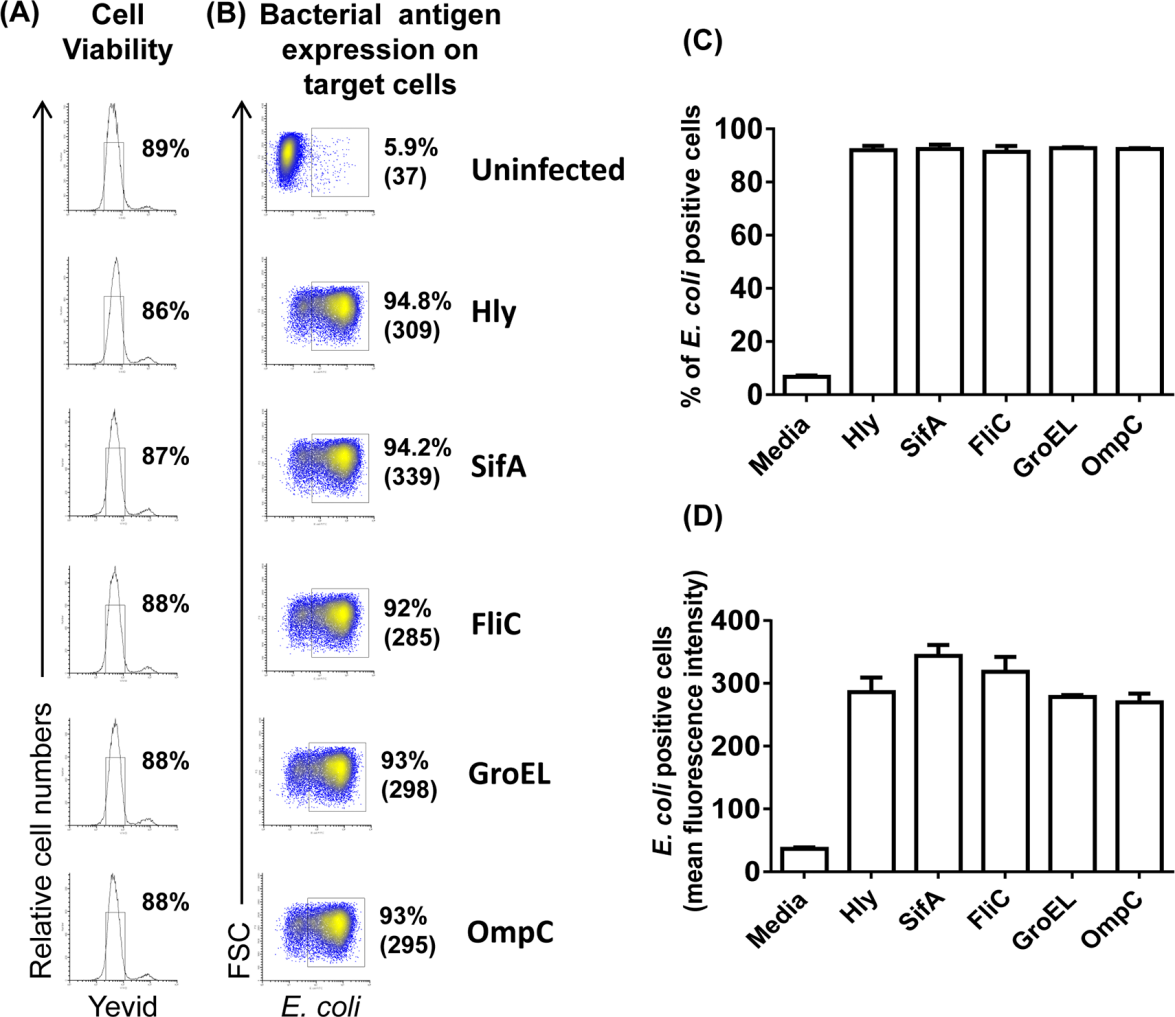
Expression of bacterial antigens on B-LCL target cells. B-LCL cells were infected with *E*. *coli* at 1:30 MOI with one of the four recombinant *E*. *coli* expressing *S*. Typhi and Hly antigens: Hly/SifA (SifA), Hly/FliC (FliC), Hly/GroEL (GroEL) and Hly/OmpC (OmpC). Uninfected B-LCLs (uninfected) and infected with recombinant *E*. *coli* expressing only Hly antigen were used as controls. The (**A**) viability and the (**B**) percentage of the *E*. *coli*-expressing cells were assessed by flow cytometry. Numbers correspond to the % of positive cells in the indicated quadrant in each histogram followed by mean fluorescence intensity (MFI) of positive cells (in parenthesis). Cumulative data of (**C**) % and (**D**) mean fluorescence intensity (MFI) were detected by using anti-*E*. *coli* antibody as described in Methods. Average of 3 independent experiments.

We next evaluated the effect of the recombinant *E*. *coli* infection on B-LCL viability. Briefly, we assessed cell viability by measuring the levels of Yevid viability staining on 2-hour-*E*. *coli* infected B-LCLs that have been rested overnight in the presence of gentamicin. As shown in **Fig 2A**, regardless of the protein being expressed, the infection did not adversely affect the viability of *E*. *coli*-infected B-LCLs. After infection, the percentage of live cells in cultures with recombinant *E*. *coli* was comparable to control cultures with media only (uninfected). By using the same experimental conditions as for determinations of cell viability, we also detected the expression of bacterial antigens on B-LCLs. Similarly to the viability, regardless of the type of protein being expressed in the recombinant *E*. *coli*, we found similar levels of *E*. *coli*-expressing cells as assessed by surface staining with anti-*E*. *coli* antigen polyclonal antibody using flow cytometry (**Fig 2B–2D**).

### Hly functionality

**Fig 3:**
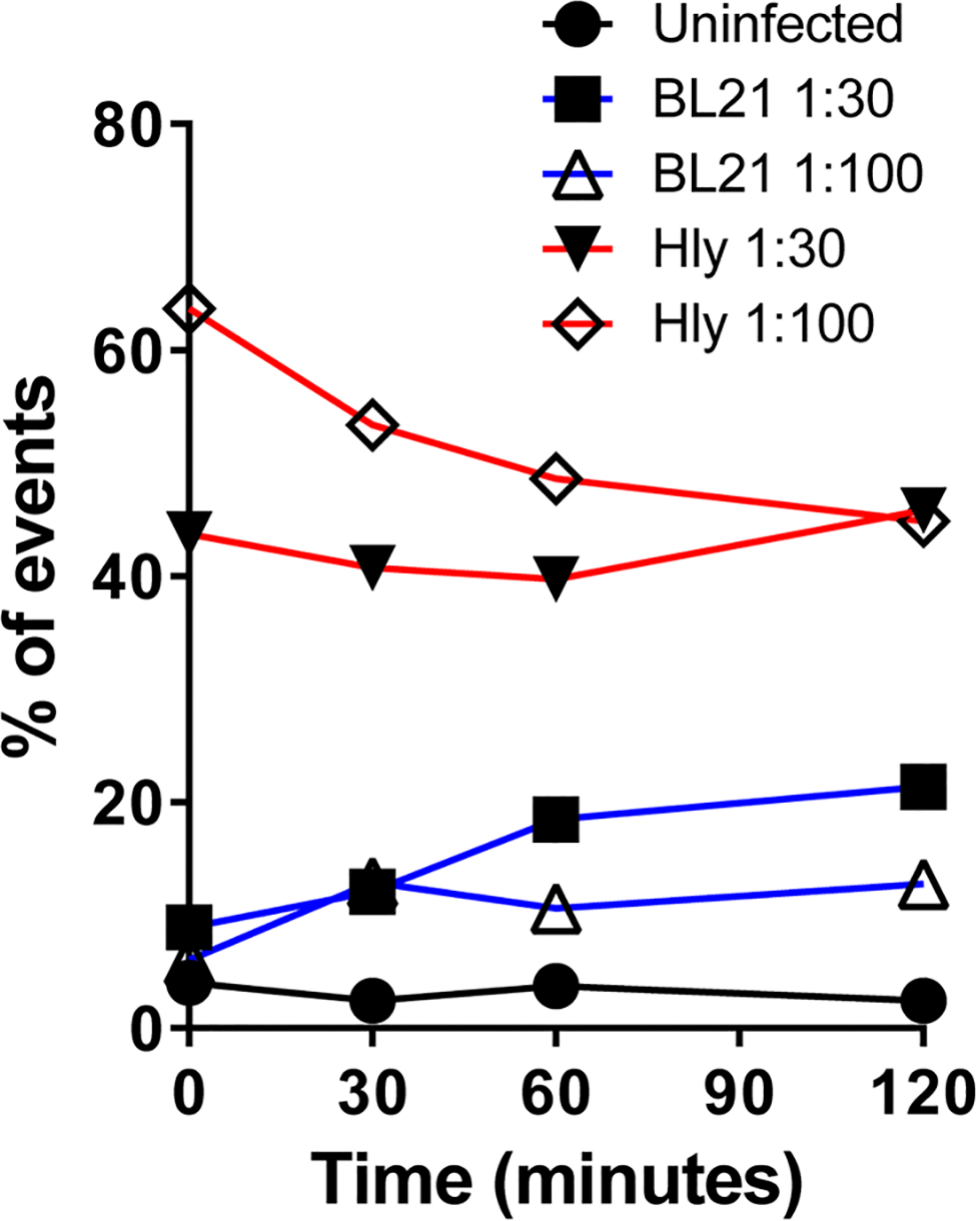
Ability of the *Hly* gene to improve antigen processing. B-LCL cells were infected with either *E*. *coli* strain BL21 (BL21) or its *Hly*-recombinant *E*. *coli* (Hly) counterpart at 1:30 or 1:100 multiplicity of infection (MOI). After 2 hours, the cells were washed and exposed to gentamicin for an additional 2 hours to kill and detach extracellular bacteria. After further washings, the ability of the *Hly* gene to improve antigen processing was assessed by detecting *E*. *coli* antigens at the B-cell surface over time by flow cytometry (up to 120 minutes). Cells exposed to media only were used as control (uninfected).

As described above, we reasoned that Hly should promote the phagosomal escape of bacterial antigens thereby improving MHC class I processing of *S*. Typhi antigens presented by B-LCLs and hence recall immune responses from both CD4^+^ and CD8^+^ primed T cells [8, 26, 27, 29, 30, 39, 47]. To test this assumption, *Hly*-recombinant *E*. *coli* strain BL21, or wild type *E*. *coli* strain BL21 were used to infect B-LCL cells. Cells were infected for 2 hours using two different multiplicity of infection (MOI, 1:30 and 1:100). After 2 hours, cells were collected, washed to remove extracellular bacteria and cultured in the presence of gentamicin for 2 additional hours. Thus, the ability to detect *E*. *coli* proteins in B-LCL infected cells was assessed over time by flow cytometry (up to 120 minutes) using polyclonal anti-*E*. *coli* antibodies. As shown in **Fig 3**, at all-time points evaluated, we observed higher expression of *E*. *coli* antigens on B-LCL cells infected with the recombinant *E*. *coli* strain expressing Hly as compared to the wild-type *E*. *coli* strain. Thus, the *hly* gene appears functional. These results are very significant since based on our previous study [48], we expect to see background responses against the vector itself (*E*. *coli* antigens). Further antigen expression might help to better discriminate T-cell responses to *S*. Typhi antigens from those directed to *E*. *coli* antigens.

### Identification of *S*. Typhi proteins that are targets of T-cells following oral immunization with Ty21a

**Fig 4:**
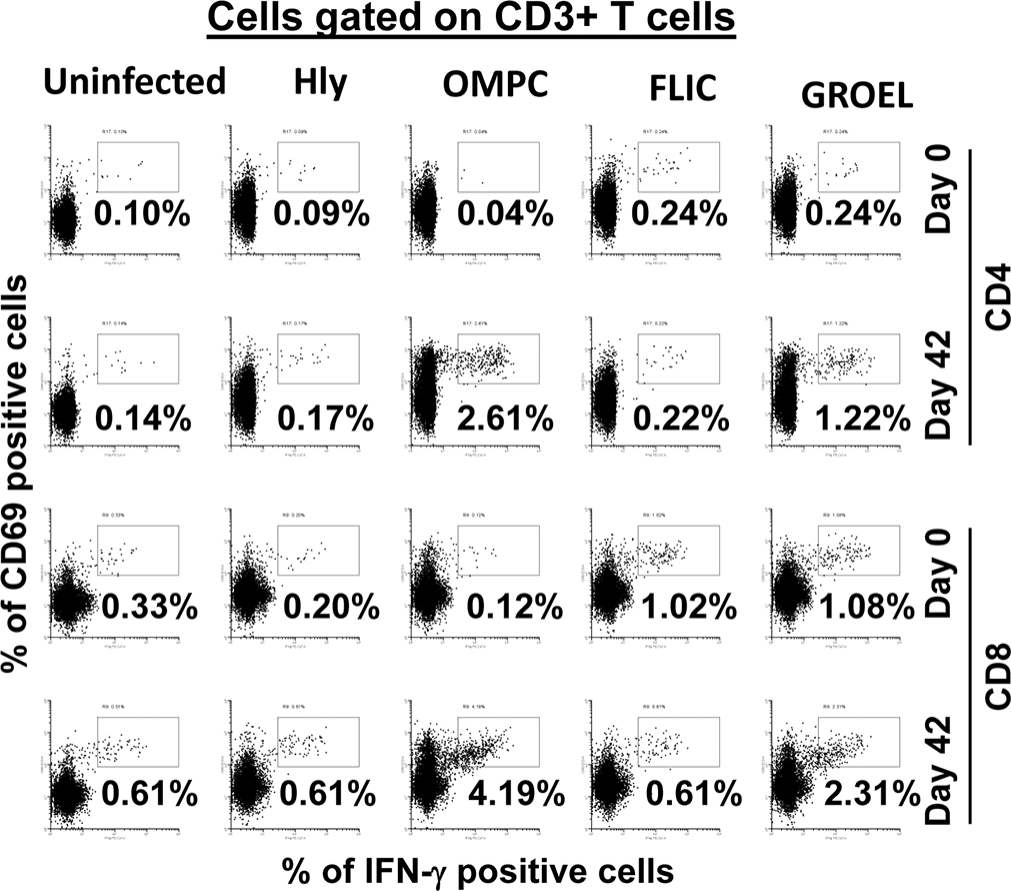
Antigen presentation of *S*. Typhi proteins by targets infected with recombinant *E*. *coli*. *Ex vivo* PBMC from a volunteer collected before (day 0) and 42 days after immunization were co-cultured for 16–18 hrs. with autologous B-LCL targets infected at 1:30 MOI with one of the four recombinant *E*. *coli* expressing *S*. Typhi/Hly (*Hly*/*SifA* (SifA), *Hly*/*FliC* (FliC), *Hly*/*GroEL* (GroEL) and *Hly*/*OmpC* (OmpC)) or only *Hly* (control) proteins. After incubation, cells were stained and the ability of the PBMC to express IFN-γ was analyzed by flow cytometry. CD4^+^ and CD8^+^ T cells were evaluated. Numbers represent the percentage of positive cells. The data of a representative volunteer are shown.

**Fig 5:**
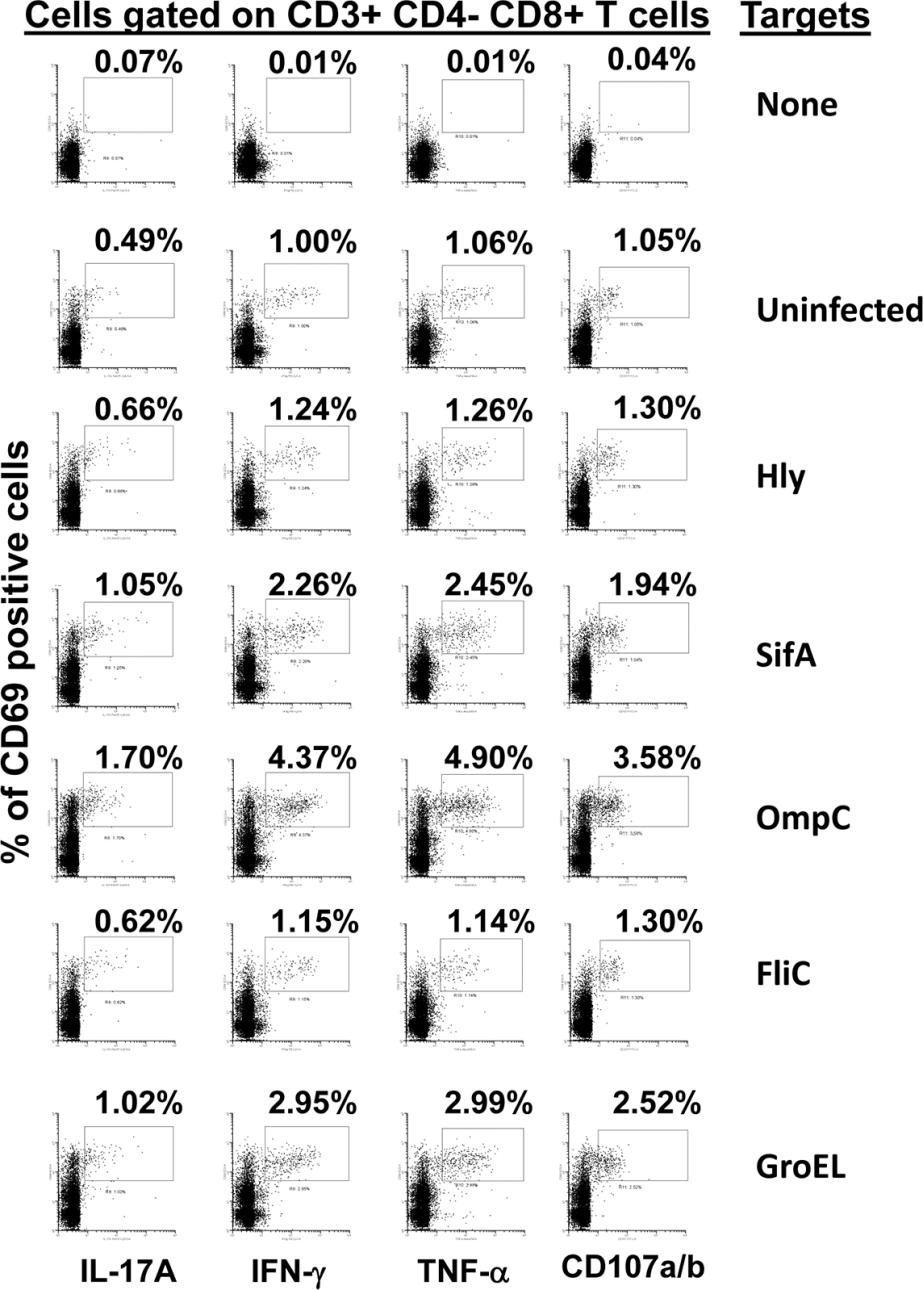
CD8+ T cell responses to *S*. Typhi proteins presented by targets infected with recombinant *E*. *coli*. *Ex vivo* PBMC from a volunteer collected 42 days after immunization were co-cultured for 16–18 hrs. with autologous B-LCL targets infected at 1:30 MOI with one of the four recombinant *E*. *coli* expressing *S*. Typhi/Hly (*Hly*/*SifA* (SifA), *Hly*/*FliC* (FliC), *Hly*/*GroEL* (GroEL) and *Hly*/*OmpC* (OmpC)) or only *Hly* (control) proteins. After incubation, cells were stained and the ability of the PBMC to express one or more cytokines (IL-17A, IFN-γ and TNF-α) and/or CD107a/b molecules was evaluated by flow cytometry. Shown are the CD8^+^ T cell responses from a representative volunteer. Numbers represent the percentage of positive cells.

**Fig 6:**
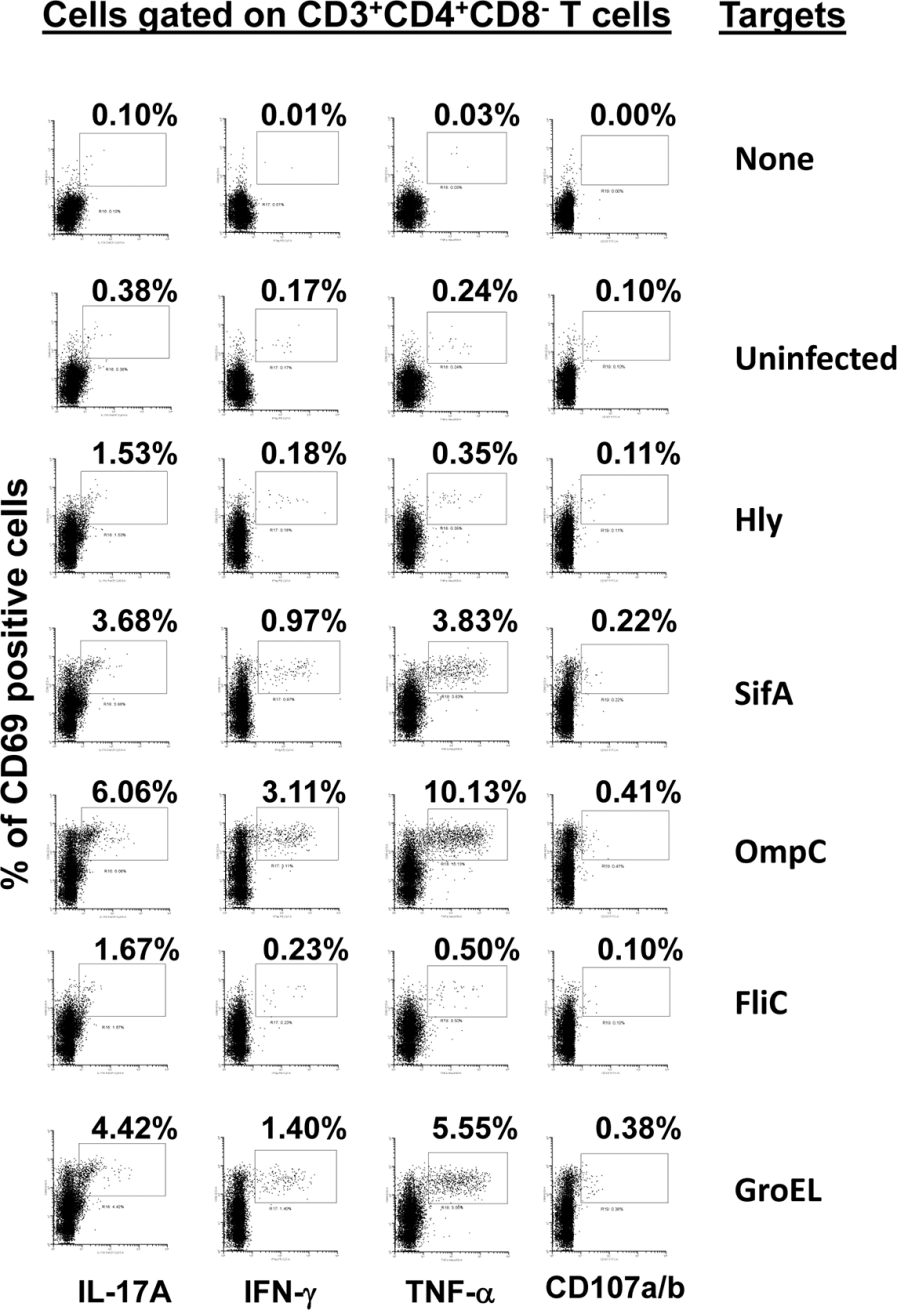
CD4+ T cell responses to *S*. Typhi proteins presented by targets infected with recombinant *E*. *coli*. *Ex vivo* PBMC from a volunteer collected 42 days after immunization were co-cultured for 16–18 hrs. with autologous B-LCL targets infected at 1:30 MOI with one of the four recombinant *E*. *coli* expressing *S*. Typhi/Hly (*Hly*/*SifA* (SifA), *Hly*/*FliC* (FliC), *Hly*/*GroEL* (GroEL) and *Hly*/*OmpC* (OmpC)) or only *Hly* (control) proteins. After incubation, cells were stained and the ability of the PBMC to express one or more cytokines (IL-17A, IFN-γ and TNF-α) and/or CD107a/b molecules was evaluated by flow cytometry. Shown are the CD4+ T cell responses from a representative volunteer. Numbers represent the percentage of positive cells.

**Fig 7:**
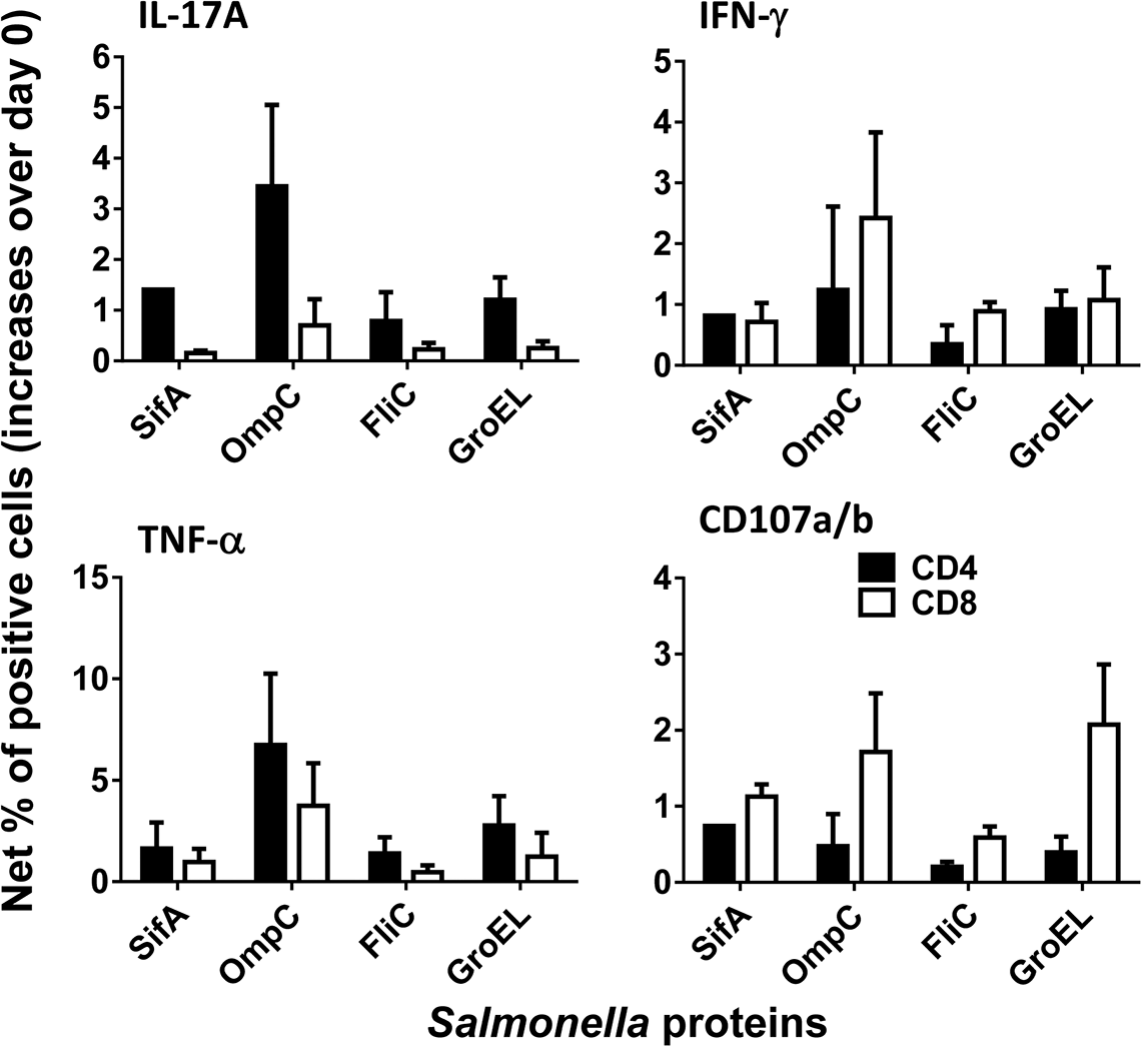
Percentage of T cell subsets specific to any *S*. Typhi protein. *Ex vivo* PBMC from 7 volunteers collected before and 42 days after immunization were co-cultured for 16–18 hrs. with autologous B-LCL targets infected at 1:30 MOI with one of the four recombinant *E*. *coli* expressing *S*. Typhi and *Hly* genes: *Hly*/*SifA* (SifA), *Hly*/*FliC* (FliC), *Hly*/*GroEL* (GroEL) and *Hly*/*OmpC* (OmpC). After incubation, cells were stained and the ability of the PBMC to express one or more cytokines (IL-17A, IFN-γ and TNF-α) and/or CD107a/b molecules was analyzed by flow cytometry. Two T cell subset responses (i.e., CD4^+^ and CD8^+^ T cells) were evaluated. Net responses were calculated by subtracting the T cell responses to B-LCLs infected with recombinant *E*. *coli* expressing *S*. Typhi/Hly antigens from the responses to the controls (B-LCL expressing *Hly* only). Increases over day 0 were calculated by subtracting the net responses of the PBMC collected 42 days after immunization from the net responses of PBMC collected before immunization. Bars represent mean ± SE.

**Fig 8:**
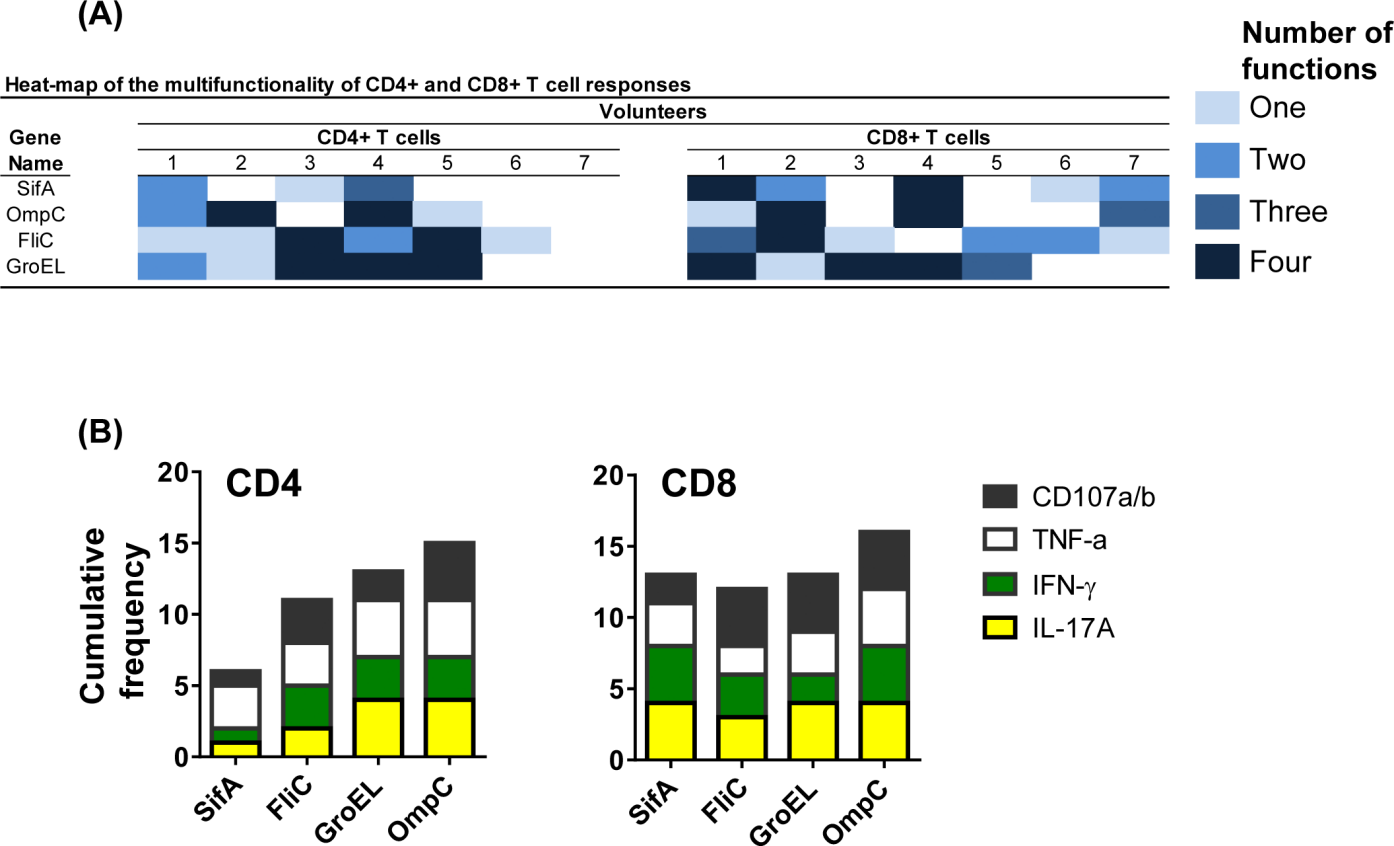
Volunteer responses to *S*. Typhi proteins. *Ex vivo* PBMC from 7 immunized volunteers collected before (day 0) and 42 days after immunization were co-cultured for 16–18 hrs. with autologous B-LCL targets infected at an 1:30 MOI with one of the four recombinant *E*. *coli* expressing *S*. Typhi and *Hly* antigens: *Hly*/*SifA* (SifA), *Hly*/*FliC* (FliC), *Hly*/*GroEL* (GroEL) and *Hly*/*OmpC* (OmpC). After incubation, cells were stained and the ability of the PBMC to express one or more cytokines (IL-17A, IFN-γ and TNF-α) and/or CD107a/b molecules was analyzed by flow cytometry. Two T cell subset responses (i.e., CD4^+^ and CD8^+^ T cells) were evaluated. (**A**) Heat-map of the multifunctionality of CD4^+^ and CD8^+^ T cells based on expression of cytokines and CD107a/b antigens. Percentages correspond to the net responses calculated by subtracting the T cell responses to B-LCLs infected with recombinant *E*. *coli* expressing *S*. Typhi/Hly proteins from the responses to the controls (B-LCL expressing *Hly* only). Volunteers were considered responders if the net responses of the PBMC collected 42 days after immunization were greater than 0.1 from the net responses of PBMC collected before immunization. (**B**) Cumulative frequency of responders to any functional test.

In order to demonstrate the feasibility of using an *E*. *coli* expression system to uncover the antigen specificity of T-cells, PBMC obtained before and 42 days after immunization were exposed to autologous B-LCL infected with recombinant *E*. *coli* expressing Hly only or co-expressing one of the four *Salmonella* gene products: Hly/SifA, Hly/OmpC, Hly/FliC and Hly/GroEL. Specifically, we studied the ability of *ex-vivo* PBMC from seven Ty21a-immunized volunteers to express IL-17A, IFN-γ and TNF-α cytokines and/or CD107a and b molecules against autologous infected targets. T-cell responses (i.e., CD4^+^ and CD8^+^ T-cells) were evaluated by multichromatic flow cytometry using a 10-color surface/intracellular staining panel. Unstimulated and *Staphylococcus* enterotoxin B (SEB)-stimulated effector cells were used as negative and positive controls, respectively. We observed that the pattern of response to individual *S*. Typhi proteins was variable, with some individuals responding to all four proteins while others were responding to only one or two proteins. We also observed differential CD4^+^ and CD8^+^ T cells responses to the *S*. Typhi proteins (**Figs 4–7**). In six individuals, although the magnitude of responses varied considerably, both CD4^+^ and CD8^+^ T cells responded to at least one protein. In one individual, we were unable to detect CD4^+^ T cells responses to any of the four protein, but CD8^+^ T cells responded to 3 out of 4 proteins tested (**Fig 8**). Representative responses from selected volunteers are shown in **Figs 4–6.**

### Multifunctional of CD4^+^ T-cells and CD8^+^ T- cells

Because previous results from our group have shown that multifunctional T-cells might contribute to *S*. Typhi immunity [30, 34, 49], we then investigated the multi-functionality patterns of CD4^+^ T-cells and CD8^+^ T-cells after exposure to infected B-LCL infected with recombinant *E*. *coli*. We measured simultaneously four T-cell functions (i.e., expression of IL-17A, IFN-γ and TNF-α cytokines, or CD107a and b molecules) by multichromatic flow cytometry using the FCOM feature of the WinList software, which provides the % of T-cells expressing each of the possible function combinations. Analyses of multiple function patterns (i.e., single, double, triple or quadruple functions) revealed that, albeit different for different proteins, both CD4^+^ T-cells and CD8^+^ T-cells were multi-functional (**Fig 8**).

### T-cell responses to exogenous proteins

**Fig 9:**
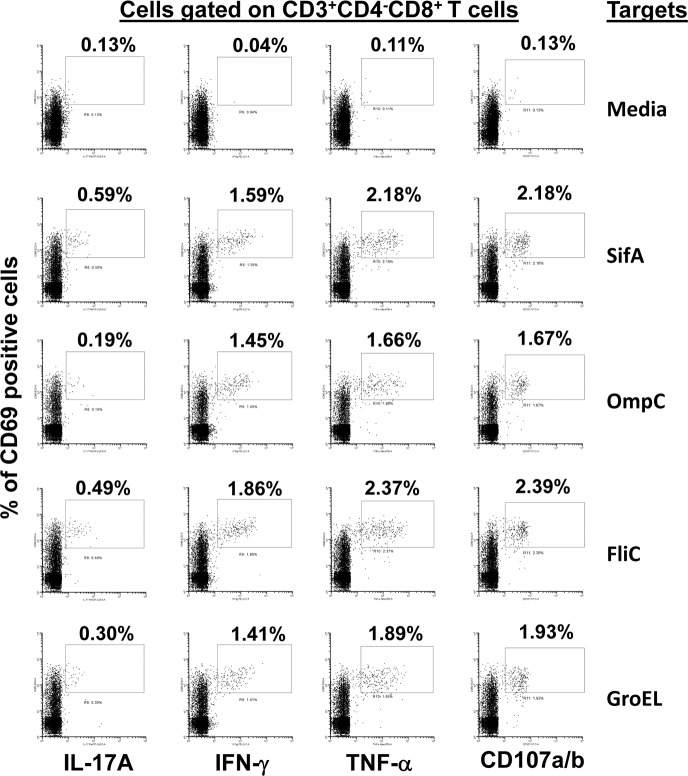
CD8+ T cell responses to *S*. Typhi proteins presented by targets exposed to one of the four recombinant *S*. Typhi proteins. *Ex vivo* PBMC from a volunteer collected 42 days after immunization were co-cultured for 16–18 hrs. with autologous B-LCL targets exposed to 0.5ug/ml with one of the four recombinant *S*. Typhi proteins: SifA, OmpC, FliC, and GroEL. Untreated B-LCL targets (media) were used as controls. After incubation, cells were stained and the ability of the PBMC to express one or more cytokines (IL-17A, IFN-γ and TNF-α) and/or CD107a/b molecules was evaluated by flow cytometry. Shown are the CD8^+^ T cell responses from a representative volunteer. Numbers represent the percentage of positive cells.

**Fig 10:**
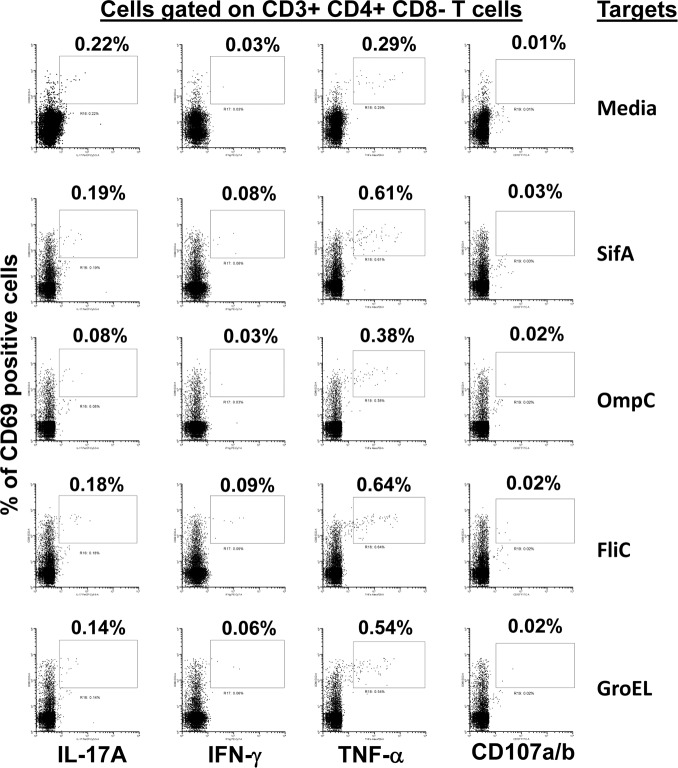
CD4+ T cell responses to *S*. Typhi proteins presented by targets exposed to one of the four recombinant *S*. Typhi proteins. *Ex vivo* PBMC from a volunteer collected 42 days after immunization were co-cultured for 16–18 hrs. with autologous B-LCL targets exposed to 0.5ug/ml with one of the four recombinant *S*. Typhi proteins: SifA, OmpC, FliC, and GroEL. Untreated B-LCL targets (media) were used as controls. After incubation, cells were stained and the ability of the PBMC to express one or more cytokines (IL-17A, IFN-γ and TNF-α) and/or CD107a/b molecules was evaluated by flow cytometry. Shown are the CD4^+^ T cell responses from a representative volunteer. Numbers represent the percentage of positive cells.

**Fig 11:**
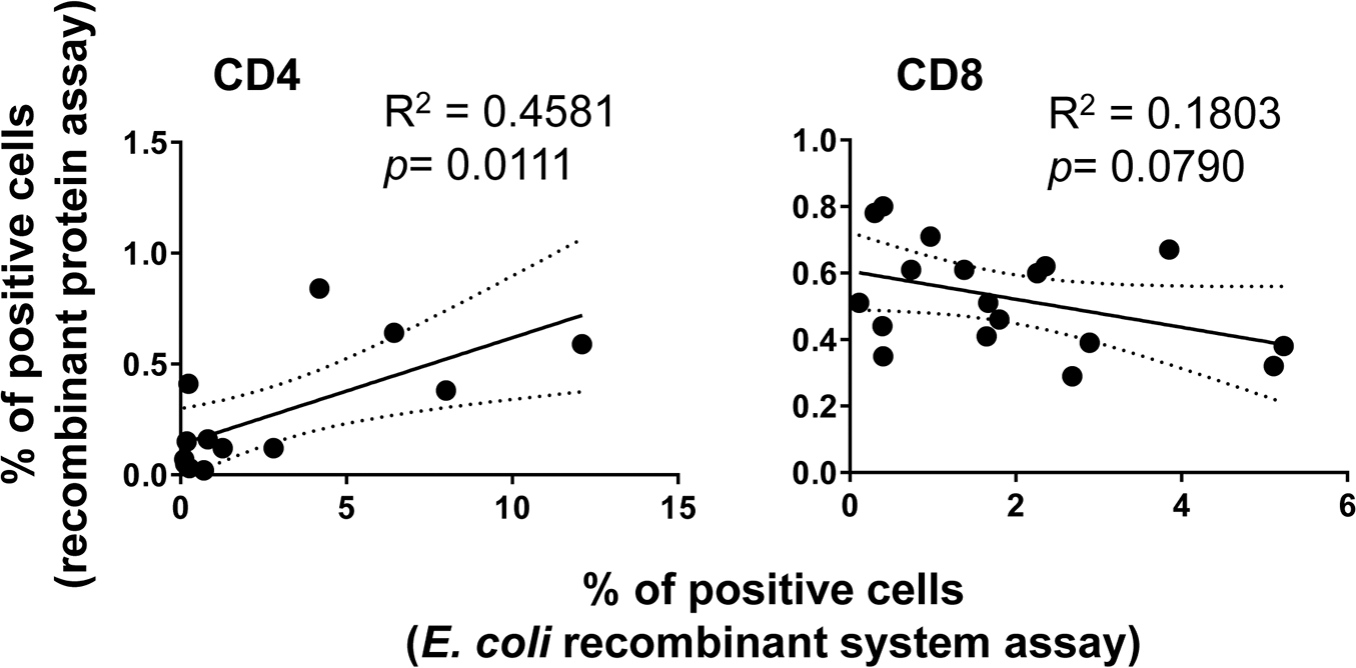
Correlation between T cell subset responses to B-LCL targets exposed to recombinant *S*. Typhi proteins or infected with recombinant *E*. *coli* expressing *S*. Typhi proteins. *Ex vivo* PBMC were analyzed as described in Figs 1 & 2. Shown are the correlation between T cell subset responses to either of the four proteins (SifA, FliC, GroEL and OmpC) after stimulation by B-LCL targets exposed to recombinant *S*. Typhi proteins or infected with recombinant *E*. *coli* expressing *S*. Typhi proteins. Samples are representative of two individuals collected 42 days after vaccination. Coefficients of determination “R2” and “p” values are shown. p values of <0.05 were considered statistically significant. Dashed lines represent 95% confidence intervals.

An important hypothesis to evaluate is whether the T-cells from volunteers who responded to B-LCL cultured with recombinant *E*. *coli* expressing *Salmonella* gene products also possess the ability to respond to exogenous proteins. To this end, we next investigated whether there was an association between T-cell responses after exposure to autologous B-LCL cultured with recombinant *E*. *coli* expressing one of the four *Salmonella* gene products (Hly/SifA, Hly/OmpC, Hly/FliC and Hly/GroEL) and those T-cell responses after exposure to B-LCL cultured with individual purified recombinant proteins (SifA, OmpC, FliC, and GroEL). To this end, B-LCLs were cultured overnight with purified SifA, OmpC, FliC or GroEL proteins, and used as targets for T-cells. Representative responses of CD8^+^ and CD4^+^ cells from one volunteer are shown in **Figs 9 & 10** respectively. We found that the CD4^+^ cell responses to B-LCLs cultured with exogenous proteins were significantly associated with the CD4^+^ cell responses to B-LCLs cultured with recombinant *E*. *coli* expressing one of the four *S*. Typhi gene products (**Fig 11**; *p* = 0.0111, Pearson Product Moment Correlation). However, no significant association was observed between CD8^+^ cell responses to B-LCLs cultured with recombinant *E*. *coli* expressing one of the four *Salmonella* gene products and CD8^+^ cell responses to B-LCLs cultured with exogenous recombinant proteins (**Fig 11**; *p* = 0.0790, Pearson Product Moment Correlation). On the other hand, CD8^+^ cell responses to B-LCLs cultured with exogenous proteins were consistently higher than CD4^+^ cell responses (**Figs 9 and 10 & S1 Table)**. Thus, as expected, CD4^+^ and CD8^+^ cell responses against *S*. Typhi antigens depend on the nature of the stimulant [39, 50]. These results emphasize the importance of selecting the stimuli appropriately when designing experiments aimed at evaluating CD4^+^ and CD8^+^ cell responses cell responses [8].

## Discussion

One of the characteristics that make the *E*. *coli* expression system methodology employed in this manuscript highly relevant for identifying immunogenic proteins of S. Typhi is the use of a translational research approach using human T cells and autologous APC to identify *S*. Typhi-specific T-cell immune responses. Most published methods have relied heavily on “proof-of-concept” studies performed in mice. However, *S*. Typhi is a human-restricted pathogen and there are no good animal models that faithfully recapitulate *S*. Typhi infection [51]. To partially address this shortcoming, the infection of susceptible mice with *S*. Typhimurium has been used as a model for the pathogenesis of human typhoid fever [51]. Although these murine models have provided considerable knowledge regarding host-pathogen interactions, they do not adequately recapitulate *S*. Typhi infection in humans [52]. The recent availability of full genome sequences from various *Salmonella enterica* serovars has uncovered many differences in pseudo genes which can explain, at least in part, the dissimilarities observed in the immune and other host responses to these enteric bacteria [52]. Thus, samples from human participants exposed to the licensed Ty21a oral typhoid vaccine have the potential to provide a better characterization of key *Salmonella* antigens involved in T cell responses than murine models.

The main novelty of our system is that we engineered the *hly* gene encoding the pore-forming cytoplasmic listeriolysin O (Hly) protein onto the backbone of the recombinant protein expression plasmid pET-DEST-Hly (see methods). In contrast, Higgin’s *E*. *coli* expressing system [37, 38] used two expression plasmids inside individual *E*. *coli* cells: one expressing Hly and the other expressing the recombinant protein of interest. The reason for this difference stems from our preliminary results showing that the use of two expression plasmids reduced B-LCL infectivity (**S1 Fig**). We speculate that this difference is due to the fitness cost for *E*. *coli* to maintain, replicate and propagate two plasmids instead of one. There may also have been a negative effect on bacterial cell preparations resulting from growth on media containing two selective antibiotics instead of one.

Herein, using this methodology, we found that all the tested individuals had increased T-cell responses over baseline (before immunization) to at least one of the four *S*. Typhi proteins evaluated (i.e., SifA, OmpC, FliC, and GroEL). Moreover, multifunctional CD4^+^ and CD8^+^ T cells that expressed two or more cytokines (IL-17A, IFN-γ and TNF-α) and/or CD107a/b molecules were detected. These results are particularly significant since we have previously demonstrated that these two T-cell population might play a role in controlling *Salmonella* infection [39]. These results also support previous data showing that the depletion of either CD4^+^ or CD8^+^ T-cells had impaired recall immunity to oral challenge with the virulent *S*. Typhimurium at different times after vaccination [53].

The reason underlying the observation that the responses to the different antigens are variable among the vaccinees are unclear. However, it is reasonable to speculate that this phenomenon might be due to the HLA‐haplotype variation between individuals. In fact, antigen processing, together with defined MHC genes, are known to shape the individual immune responses to a wide array of pathogens [54]. Furthermore, the differential responsiveness among the participants supports the development of multi-component vaccines by introducing many antigenic determinants into vaccine formulations. Alternatively, these results might encourage a renewed focus on whole-cell live attenuated preparations, especially since they may overcome some of the inherent weaknesses associated with sub-unit vaccines such as the need for considerable amounts of antigens and the use of adjuvants.

We also observed that the magnitude of the responses against *S*. Typhi SifA, OmpC, FliC and GroEL varied among participants. Since *S*. Typhi GroEL has a significant degree of homology with self-heat shock proteins in humans [27], these results provide additional information that T-cells can discriminate between self and foreign antigens during the immune response. Interestingly, in contrast to CD8^+^ cell responses, we found that the CD4^+^ cell responses to B-LCLs cultured with exogenous proteins were significantly associated with the CD4^+^ cell responses to B-LCLs cultured with recombinant *E*. *coli* expressing *S*. Typhi genes. These results confirm and extend previous findings from our group and others that the balance of CD4^+^ and CD8^+^ cell responses against *S*. Typhi antigens are likely to depend on the nature of the stimulant [8, 39, 50]. Indeed, previous work from our group has shown that CD4^+^ cells respond differently to soluble antigen stimulation than CD8^+^ T cells [39, 50]. Future studies will be directed to use this novel antigen discovery system to evaluate in humans the immune responses to other *S*. Typhi proteins expressed in this *E*. *coli* expression system.

Since these studies were performed in immunized volunteers, it is also important to note that the relative contributions of CD4^+^ and CD8^+^ cell responses against these *S*. Typhi proteins in protection cannot be ascertained. We are directly addressing this critical issue in separate studies in which we are evaluating whether responses to these *S*. Typhi proteins correlate with protection in volunteers who have been immunized with Ty21a and subsequently challenged with wild-type *S*. Typhi. Nevertheless, the results presented herein demonstrate the feasibility of using a novel antigen discovery platform. This system could be used, for example, to systematically assess the specificity of T-cell immune responses against the entire proteome of a human pathogen and generate a database of the repertoire of these human T-cell antigen specificities. This should help narrow down the proteins of interest which correlate with defined phenotypes (e.g., responses associated with protection in human challenge studies with *S*. Typhi, those directed to pathogenic determinants), ultimately leading to the identification of candidate vaccine antigens. Finally, these results emphasize the importance of selecting the appropriate antigens when designing experiments aimed at evaluating CD4^+^ and CD8^+^ cell responses.

## Materials and methods

### Ethics statement

The human experimentation guidelines of the US Department of Health and Human Services and those of the University of Maryland, Baltimore, were followed in the conduct of the clinical research. All blood specimens were collected from volunteers who participated in the University of Maryland Institutional Review Board approved protocol number HP-00040022 that authorized the collection of blood samples from healthy volunteers for the studies included in this manuscript. Volunteers were explained the purpose and possible consequences of participating in this study and gave informed, signed consent before the blood draws. This protocol has been conducted in accordance with the ethical standards laid down in the 1964 Declaration of Helsinki and the principles of the International Conference on Harmonization Good Clinical Practice guidelines [55].

### Participants

Seven healthy adult volunteers, 20–50 (39 ± 10) years old, recruited from the Baltimore-Washington area and the University of Maryland at Baltimore campus, participated in this study. They were immunized with four spaced doses of 2–6 x 10^9^ CFU of oral live attenuated Ty21a at an interval of 48 hours between doses [12, 56]. Blood collection was performed before and 42 days after Ty21a immunization. Peripheral blood mononuclear cells (PBMC) were isolated from the blood by density gradient centrifugation and cryopreserved in liquid N_2_ following standard techniques [28]. These PBMC were used *ex vivo* as effector cells or to prepare the target cells.

### Gateway cloning vectors

The entire *S*. Typhi strain Ty2 ORFeome was constructed as a part of the former National Institute of Allergy and Infectious Disease (NIAID)-funded Pathogen Functional Genomics Resource Center (PFGRC). The PFGRC provided a library of entry clones by cloning 3,381 ORFs, out of the 4,323 ORFs predicted in the Ty2 genome (78%), into the pDONR 221 vector using methods described in Peterson *et al*. [57]. Entry clones can then be shuttled into a variety of destination vectors using *in vitro* site-specific recombination (http://www.ncbi.nlm.nih.gov/pubmed/11076863). For expression of the *S*. Typhi proteins, we engineered the pET161-DEST destination vector (Invitrogen, Carlsbad, CA). The pET161-DEST, a commercial plasmid from Invitrogen, is a robust *E*. *coli* destination vector that has been used for several years and carries many desirable characteristics for this study. Gene expression is under the tight control of the T7 promoter and *lac* repressor, and an optimized ribosome binding site is provided by the inserted gene. The vector contains the V5 and His-tag epitopes downstream of the *att*R sites into which inserts from entry clones were recombined resulting in the addition of these tags as C-terminal fusions to the *S*. Typhi protein. These tags were used to monitor recombinant protein expression and, if necessary, for protein purification. pET161-DEST also contains the ccdB and chloramphenicol (Cm^R^) genes which help the recovery of correct recombination products after the LR recombinase reaction via selection on ampicillin agar plates. We also inserted the *hly* gene coding for the cytoplasmic Hly protein from *L*. *monocytogenes* into the *Bgl*II restriction site. The *hly* gene was PCR-amplified with a His-tag at the 3’end of the reverse primer, and a *BgI*ll site was added to the forward 5’ end, and reverse 3’ end of each primer, respectively (sense: GCGCAGATCTAGCAAGCATATAATATTGCG, anti-sense: GCGCAGATCTTTAGTGATGGTGATGGTGATGTTCGATTGGATTATCTAC). The resulting vector pET-DEST-Hly was verified by Sanger sequencing. It carries a T7 promoter and the lac operator preceding the gene to be cloned; the cloning site harbors attR1 and attR2 site for cloning from entry clones through the LR clonase reaction (see below). The plasmid was transformed into One Shot ccdB survival competent cells (Invitrogen).

### Cloning of S. Typhi proteins from entry clones to pET-DEST-Hly for protein expression

Entry clone glycerol stocks were streaked on LB agar with 50 μg/ml kanamycin and incubated at 37°C overnight. Single colonies were incubated overnight at 37°C in 4ml LB broth with 50 μg/ml kanamycin. Then the entry clone plasmid was extracted using the QIA prep kit (Qiagen, Valencia, CA) and DNA concentration measured using a NanoDrop instrument (Thermo Scientific, Waltham, MA). The pET-DEST-Hly glycerol stock was streaked on LB agar plates with 100 μg/ml carbenicillin and incubated at 37°C. After overnight incubation, single colonies were isolated, incubated overnight in 50 ml LB broth with 100 μg/ml carbenicillin at 37°C and the destination plasmid was extracted with the HiSpeed plasmid preparation kit (Qiagen). DNA concentration was measured by NanoDrop. We chose to evaluate four *S*. Typhi proteins: SifA, FliC, GroEL, and OmpC (**Table 1**). Each of these four *S*. Typhi proteins was then transferred *in vitro* from the entry clone plasmid into the destination plasmid through LR clonase reactions (Gateway LR clonase II Enzyme mix, Invitrogen) following the manufacturer’s protocol. We also cloned a short non-coding sequence called “pmark” that carried stop codons in all six frames of translation into pET-DEST-Hly as a negative control of protein expression. Finally, 3 μl of LR clonase reaction product was transformed into 50 μl of One Shot BL21(DE3) competent cells (Invitrogen) by chemical transformation.

### Verification of expression plasmids

Proper cloning of the target *S*. Typhi gene was confirmed by PCR amplification using the T7 forward and reverse primers and sequencing of the PCR products. The presence of the *hly* gene was verified by *BgI*ll digestion of the plasmid.

### Protein expression

A single colony from verified BL21 (DE3) expression vector clones was cultured at 37°C overnight in 2 ml LB broth with 100 μg/ml carbenicillin. Then 30 μl of the overnight culture was inoculated to 3 ml fresh LB broth with 100 μg/ml carbenicillin. After ~4h of incubation at 37°C (OD~0.6), the culture was induced for protein expression with 100 μM IPTG and incubated for an additional 2 h. Bacteria were then spun down at 2,800 rpm for 10 min and the supernatant discarded. *E*. *coli* were lysed with of 0.3 μg/ml lysozyme in Tris-HCl buffer, pH 7.5 (Thermo Scientific), then proteins were heat-denatured at 100°C for 10 minutes and evaluated on SDS-PAGE gels by Coomassie staining. Specificity of protein expression was confirmed by western blot using mouse anti-His monoclonal antibodies (Sigma, St. Louis, MO), and rabbit anti-mouse IgG conjugated to horseradish peroxidase (HRP) (Sigma) as secondary antibodies; as well as rabbit anti-Listeriolysin polyclonal antibodies (Abcam, Cambridge, MA, USA), and goat anti-rabbit IgG-HRP (Millipore, Billerica, MA) as secondary antibodies. His-tagged Annexin 3 (A3) protein (Abcam) was used as positive control for antibody detection.

### Target/stimulator cells

PBMC obtained from Ty21a vaccines before immunization were used to generate autologous Epstein-Barr Virus (EBV)-transformed B-LCLs as previously described [27, 31]. Briefly, B-LCLs were established by using B95-8 cell line (ATCC# CRL1612) supernatants as the EBV source. After transformation, B-LCL were maintained in culture in RPMI 1640 (Gibco, Grand Island, New York) supplemented with 100 U/ml penicillin, 100 μg/ml streptomycin, 50 μg/ml gentamicin, 2 mM L-glutamine, 2.5 mM sodium pyruvate, 10 mM HEPES buffer and 10% heat-inactivated fetal bovine serum (R10) or cryopreserved until used in the experiments.

### Infection of target/stimulator cells by E. coli

Target/stimulator cells were infected as previously described [27–30, 39, 47, 48] with slight modifications. Briefly, target cells were infected by incubation in RPMI (without antibiotics) at 37°C for 2 hours with any of the recombinant *E*. *coli* strains at 1:30 or 1:100 multiplicity of infection (MOI). After incubation, cells were washed and incubated for an additional 2 or 16–18 hours (overnight) in complete R10 containing gentamicin (100 μg/ml) to kill extracellular and/or to detach cell-bound bacteria. For co-culture experiments, targets were then gamma-irradiated (6,000 rads), surface stained with anti-CD45, a marker abundantly expressed on the surface of hematopoietic cells [58], and used as stimulators. To confirm *E*. *coli* infection, aliquots of targets were surface stained with rabbit anti-*E*. *coli* antigen polyclonal antibody (1:1000, Abcam).

### Exogenous proteins

Four *Salmonella* purified proteins were tested: SifA, FliC, GroEL, and OmpC (**Table 1**). The region encoding residues 53–450 of FliC were subcloned from *S*. Typhi ISP1820 into pET15b. The plasmid was used to transform *E*. *coli* Tuner (DE3) for overexpression after being induced with IPTG. The overexpressed protein was purified by standard immobilized metal affinity column chromatography (IMAC) methods. OmpC was purified based on the protocol of Kumar and Krishnaswamy [59]. The sequence encoding OmpC from *S*. Typhi ISP1820 was subcloned into pET15b, which was used to transform *E*. *coli* Tuner (DE3). Overexpression was induced using IPTG, and the OmpC was purified from inclusion bodies using IMAC after solubilizing in buffer containing urea. Refolding was then allowed to occur in 50 mM Tris, pH 8.5, 0.1 M NaCl, 10% (v/v) glycerol and 0.2% (v/v) polyoxyethelene-9-lauryl ether. The sequence for GroEL was introduced into pBAD TOPO TA such that it was in-frame. The protein was overexpressed by the addition of arabinose, and the HT-GroEL purified initially by standard IMAC, which provides good yields at high purity. Removal of contaminating peptides could be achieved by adding acetone to 45% (v/v) to precipitate the GroEl which could then be collected by centrifugation followed by solubilizing using PBS (10 mM phosphate, pH 7.4, 150 mM NaCl). The sequence encoding SifA from *Salmonella* Typhi ISP1820 was subcloned into pET15b and used to transform *E*. *coli* c-41 (DE3). The bacteria were grown to an absorbance of about 0.8 at 600 nm, protein expression induced with IPTG, and the cultures immediately moved to 20°C for overnight growth. The bacteria were then lysed and the SifA purified by standard IMAC methods.

### Loading of target/stimulator cells with exogenous proteins

B-LCLs were incubated overnight in 24-well plates at a density of 2 x 10^6^/ml/well in the absence or presence of 1 μg/ml of each of the purified proteins at 37°C in a 5% CO_2_ atmosphere. After incubation, cells were washed and used as stimulators for T-cells.

### Monoclonal antibodies for surface and intracellular staining

Cells were stained with monoclonal antibodies (mAbs) to CD69 (clone TPI-55-3) (Beckman-Coulter, Miami, FL), CD4 (clone RPA-T4), CD8 (clone HIT8a), CD107a and b (clones H4A3 and H4B4 respectively), interferon (IFN)-γ (clone B27), tumor necrosis factor (TNF)-α (clone MAb11) (BD Pharmingen, San Diego, CA, USA), CD14 (clone TuK4), CD19 (clone SJ25-C1), CD45 (clone H130) (Invitrogen), interleukin (IL)-17A (clone eBio64DEC17) (eBioscience, San Diego, CA), and CD3 (clone OKT3)(Biolegend, San Diego, CA). Antibodies conjugated to the following fluorochromes were used in these studies: Fluorescein isothiocyanate (FITC), PE-Cy5.5, PE-Cy7, V450, Brilliant Violet (BV)570, BV650, Energy Coupled Dye or PE-Texas-Red conjugate (ECD), allophycocyanin (APC)-Alexa 700 and Quantum Dot (QD) 800.

### Effector cells and co-culture

*Ex vivo* PBMC from immunized volunteers collected before and 42 days after immunization were used as effectors as previously described [48]. Briefly, PBMC were co-cultured with autologous B-LCL cells at an effector to stimulator cell ratio of 5:1 in the presence of mAbs to CD107a and CD107b (15 μl of each/1 x 10^6^ cells in 500 μl of R10 medium). The CD107 a and b antibodies were used to measure degranulation, a mechanism essential for the killing of *S*. infected targets by the cytotoxic CD8^+^ cells [60]. PBMC cultured with uninfected target cells or *Staphylococcus* enterotoxin B (SEB) (10 μg/ml, Sigma) were used as negative and positive controls, respectively. After ~2 hours of stimulation, protein transport blockers, Monensin (1 μg/ml, Sigma) and brefeldin-A (BFA) (2 μg/ml, Sigma), were added to the co-culture. After overnight (16–18 hours) incubation, cells were harvested, stained with a dead-cell discriminator, yellow fluorescent viability dye (Yevid, Invitrogen)[61, 62], followed by surface staining with mAbs against surface antigens (CD3, CD4, CD8, CD14, and CD19) and fixation and permeabilization with Fix & Perm cell buffers (Invitrogen, Carlsbad, CA). Cells were then stained intracellularly for IFN-γ, TNF-α, IL-17A and CD69. Finally, cells were fixed and analyzed by flow cytometry on an LSR-II instrument (BD Biosciences). Data were analyzed with WinList 7.0 (Verity Software House, Topsham, ME). Lymphocytes were gated based on their scatter characteristics. Single lymphocytes were gated based on forward scatter height vs. forward scatter area. A “dump” channel was used to eliminate dead cells (Yevid^+^) as well as macrophages/monocytes (CD14^+^), B lymphocytes (CD19^+^) and targets (CD45^+^) from analysis. Additional gating on CD3, CD4, and CD8 was performed to identify cytokine-producing (IFN-γ, TNF-α and IL-17A) and CD107 expressing T cell subsets. Net responses were calculated by subtracting the number of positive events of the experimental (*Salmonella-*Hly proteins) from the negative control (Hly only). Functional responses were considered specific for *S*. Typhi if the differential in the number of positive and negative events between experimental (*Salmonella-*Hly proteins) and negative control (Hly only) cultures were significantly increased (*P* < 0.01) using *Z*-test. Volunteers were considered responders if the net responses from the PBMC collected 42 days after immunization were greater than 0.1% from the net responses of PBMC collected before immunization.

### Statistical analysis

All statistical tests were performed using Prism software (version 7, GraphPad Software, La Jolla, CA). Comparisons between two groups were carried out by Student’s t tests. Correlation analysis was achieved by Pearson Product Moment Correlation tests. *P* values <0.05 were considered significant.

## Supporting information

S1 Table T-cell responses to individual S. Typhi proteins. (TIF)

S1 Fig Comparison between the use of one and two expression plasmids inside individual recombinant *E. coli*. B-LCL cells were infected with *E*. *coli* expressing one (Hly/pmark or Hly/*Salmonella* protein) or two plasmids (Hly only or Hly and *S*. Typhi protein) at 1:30 MOI. *S*. Typhi proteins were FliC, GroEL and OmpC. Uninfected B-LCLs and B-LCLs infected with recombinant *E*. *coli* expressing only Hly antigen were used as controls. The percentage of the *E*. *coli*-antigen expressing B-LCLs were assessed by flow cytometry using anti-*E*. *coli* antibody as described in Methods. Shown are the average of 3 independent experiments. *p* values of <0.05 were considered statistically significant.
(TIF)
