# Supplementary figures and images for: Use of a novel antigen expressing system to study the Salmonella enterica serovar Typhi protein recognition by T cells
Source: PLoS Negl Trop Dis. 2017 Sep 5;11(9):e0005912. doi: 10.1371/journal.pntd.0005912 (PMC5600385; doi:10.1371/journal.pntd.0005912)

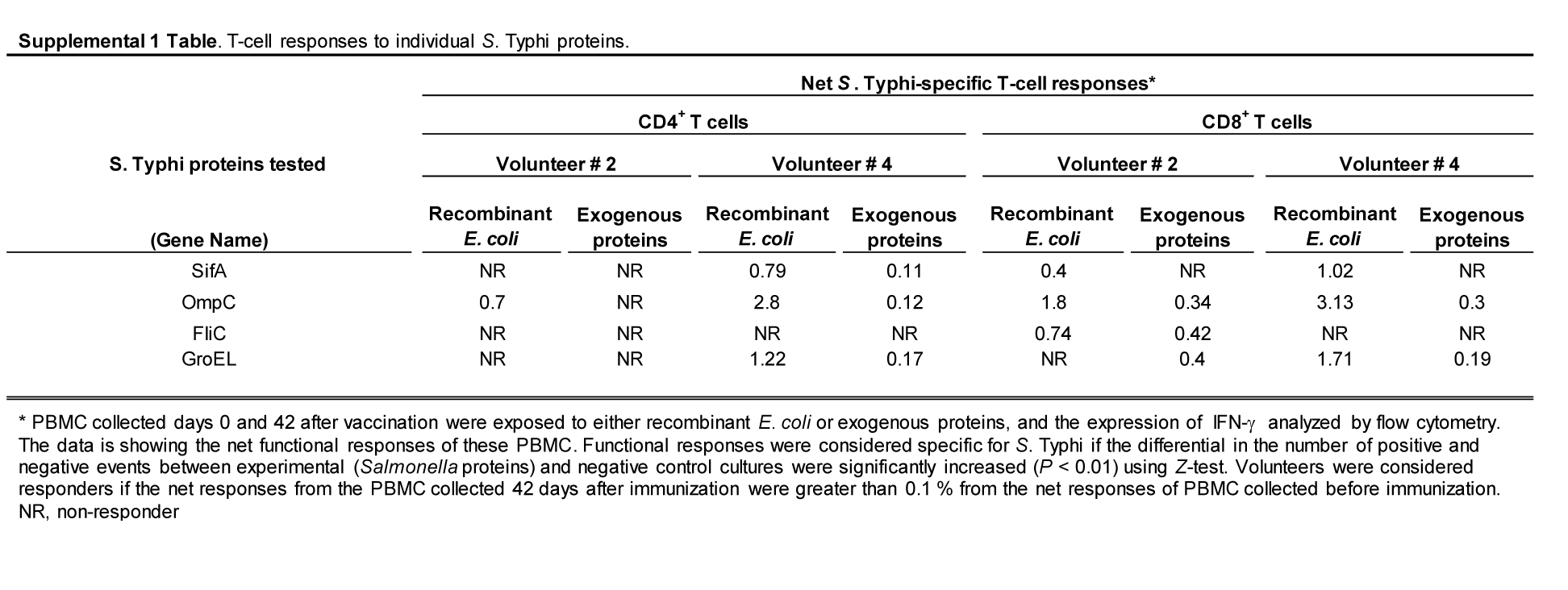

Supplement: S1 Table — (TIF) [file pntd.0005912.s001.tif]

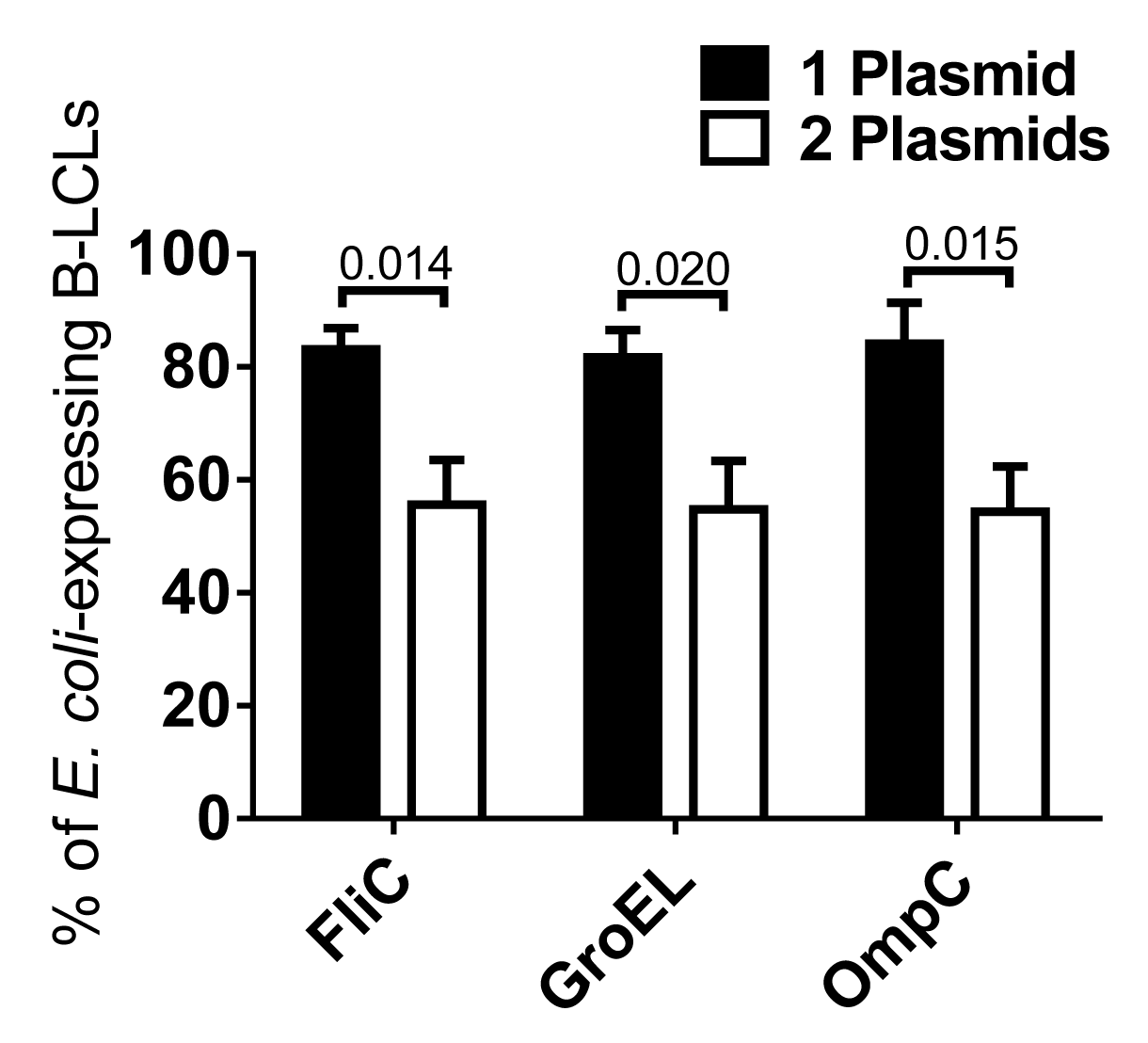

Supplement: S1 Fig — B-LCL cells were infected with E. coli expressing one (Hly/pmark or Hly/Salmonella protein) or two plasmids (Hly only or Hly and S. Typhi protein) at 1:30 MOI. S. Typhi proteins were FliC, GroEL and OmpC. Uninfected B-LCLs and B-LCLs infected with recombinant E. coli expressing only Hly antigen were used as controls. The percentage of the E. coli-antigen expressing B-LCLs were assessed by flow cytometry using anti-E. coli antibody as described in Methods. Shown are the average of 3 independent experiments. p values of <0.05 were considered statistically significant. (TIF) [file pntd.0005912.s002.tif]
